# Genomic data is missing for many highly invasive species, restricting our preparedness for escalating incursion rates

**DOI:** 10.1038/s41598-022-17937-y

**Published:** 2022-08-17

**Authors:** Paige Matheson, Angela McGaughran

**Affiliations:** Te Aka Mātuatua - School of Science, University of Waikato, Private Bag 3105, Hamilton, 3240 New Zealand

**Keywords:** Bioinformatics, Evolutionary genetics, Population genetics, Evolutionary biology, Genomics, Population genetics, Sequencing

## Abstract

Biological invasions drive environmental change, potentially threatening native biodiversity, human health, and global economies. Population genomics is an increasingly popular tool in invasion biology, improving accuracy and providing new insights into the genetic factors that underpin invasion success compared to research based on a small number of genetic loci. We examine the extent to which population genomic resources, including reference genomes, have been used or are available for invasive species research. We find that 82% of species on the International Union for Conservation of Nature “100 Worst Invasive Alien Species” list have been studied using some form of population genetic data, but just 32% of these species have been studied using population genomic data. Further, 55% of the list’s species lack a reference genome. With incursion rates escalating globally, understanding how genome-driven processes facilitate invasion is critical, but despite a promising trend of increasing uptake, “invasion genomics” is still in its infancy. We discuss how population genomic data can enhance our understanding of biological invasion and inform proactive detection and management of invasive species, and we call for more research that specifically targets this area.

## Introduction

Anthropogenic activities, such as global trade and transport over recent decades, have strongly shaped the geographic scope, frequency, and taxonomic trends of species movement beyond their natural ranges^1^. Many species introductions have historically had benign, relatively small, or even beneficial impacts—for example, providing habitat or food resources to native species or crucial ecosystem functions^2^. However, some introduced species have the potential to become ‘invasive’—that is, expand demographically and spatially and impose negative consequences in their new environment^3^. Invasive species are pervasive drivers of global change^1,4,5^, potentially altering ecosystem function^5^, introducing new ecological pressures^6^, and leading to genotype loss (e.g. via hybridisation^7^) or blurred regional distinctiveness of native biota^3^, population decline, or extinction of indigenous species^8^. The total global reported cost of invasions between 1970 and 2017 was estimated to be at least US$1.288 trillion^9^. However, the true economic effects of invasive species are difficult to quantify due to their indirect impacts (e.g. reduced plant cover, increased soil erosion, increased eutrophication^7^).

As a leading driver of environmental change, increasing attention has been dedicated towards a unified understanding of the invasion process (e.g.^10,11^). Despite this, there are significant gaps in our mechanistic conception of invasion, as well as our ability to quantify and forecast impacts caused by invasive species^11,12^. Yet, rates of biological incursion are increasing with international trade and climate change^5,13^: 37% of all globally established invasive species in the last 200 years are estimated to have been introduced after 1970^14^, and established invasive numbers per continent are predicted to increase by 36% between 2005 and 2050^15^.

Though pre-existing traits, such as broad physical tolerance and high rates of dispersal, reproduction, and growth^16,17^ are key parameters of successful invasion, certain intrinsic genetic features may make for more successful invaders^18,19^. Emerging research is identifying links between invasive potential and genomic changes (e.g.^20–22^), with alterations to gene expression, gene interaction, or genomic architecture potentially leading to a greater diet breadth (e.g.^23^), competitive advantage (e.g.^24^), and/or adaptive response to environmental change (e.g.^18^). More generally, genetic, demographic, and environmental factors interact to determine invasion success, and understanding of genetic characteristics, such as pre-adaptation and population connectivity (see Box 1 in^25^), is crucial for monitoring, managing, and mitigating the impact of invasive species.

Invasion ecology has benefited from a union with population genetic approaches (i.e. ‘invasion genetics’) for over 55 years^26^. This has resulted in broad understanding of the evolutionary processes associated with invasion, such as the general effects of bottlenecks and genetic drift on invasion success and the specific adaptive responses of some invasive species^27^. However, much invasive biology research still suffers from a lack of information around complex processes operating at the genomic level^28,29^. Moving from a ‘genetic’ (single or few loci) lens to a genome-wide (‘genomic’) one can improve analytical accuracy in some scenarios^30^. For example, the ability of mitochondrial DNA (mtDNA) to track recent invasions can be limited as this marker accumulates variation over longer timescales—for invasive mammals in particular, mtDNA can incorrectly identify an invasive populations’ country of origin compared to higher resolution genome-wide markers^31^. Such was the case for raccoons (*Procyon lotor*), which show low mtDNA variation in their invasive European range^32^, and brown rats (*Rattus norvegicus*) that have invaded New Zealand and show a European origin with mtDNA, but an admixed Asian and non-Asian ancestry using genome-wide markers^33^. In other contexts, genomic data can allow new questions to be addressed that are intractable with a small number of loci. For example, genome-wide scans in invasive populations of *Drosophila suzukii* and monkeyflower *(Mimulus guttatus*) have identified new genes that are associated with invasion routes and stress adaptation during invasion, respectively^34,35^. With respect to management, next generation sequencing technologies can facilitate proactive *community*-wide detection and identification, and ongoing monitoring programmes (e.g. in aquatic systems^36^); it can also provide targeted frameworks for eradication plans by revealing crucial information, such as dispersal patterns and population connectivity (e.g.^37^).

Recent advances in sequencing, and associated downstream analytical approaches, are thus cementing the link between genomics and invasion biology^30^ in the new field of ‘invasion genomics’. Population genomics in particular, involves the analysis of genomic patterns within and among populations to make evolutionary inferences^38^. Associated high-throughput sequencing of entire genomes or genome-wide SNPs (single nucleotide polymorphisms) for multiple individuals and populations of interest is facilitating research into population structure, demographic history, and selective processes^39,40^. In an invasive context, population genomics can be used to provide greater insights than genetic studies based on a small number of loci by accurately identifying source or high risk populations, pinpointing genomic weaknesses, studying demo-genetic factors involved in the invasion process (e.g. genetic bottlenecks, founder effects), and examining particular ‘invasive’ genes and their roles in rapid evolution^25,41^. Meanwhile, complete genomic sequences, i.e. ‘reference genomes’, provide the basis for within- and between-species insights (such as the genomic architecture of important phenotypic traits^19^), and support the development of new technologies that may be applied to pest management (e.g. targeted SNP panels or gene drives^42^).

Minimising the impact of invasive species in the Anthropocene will require a strong emphasis on proaction and prevention, and genomic data can be leveraged to support this. Reviews by Rius et al*.*^39^ and McCartney et al*.*^40^ investigated the use of next-generation sequencing techniques to study invasive species, and documented the availability of genome assemblies for species from the International Union for Conservation of Nature (IUCN) “100 of the World’s Worst Invasive Alien Species” list (‘WAS List’, hereafter), respectively. Here, we investigate the extent to which *population genomic* data has been used or is available to study globally invasive species from the WAS List, and provide an update on how many of these species currently have assembled reference genomes. We also analyse our results in a *population genetic* context, determining the number of species that have not been analysed using any form of genetic marker and marking the shift between genetic and genomic studies. We begin by illustrating a promising trend of increasing uptake of genomic research for invasive species generally before showing that, despite this, the majority of such research for WAS List species has lacked a population genomics context and genomic resources are still entirely absent for many of these species. These discouraging gaps must be addressed if we are to prepare for escalating rates of biological invasion in the future.

## Results

### Genomics of invasive species research is escalating

**Figure 1:**
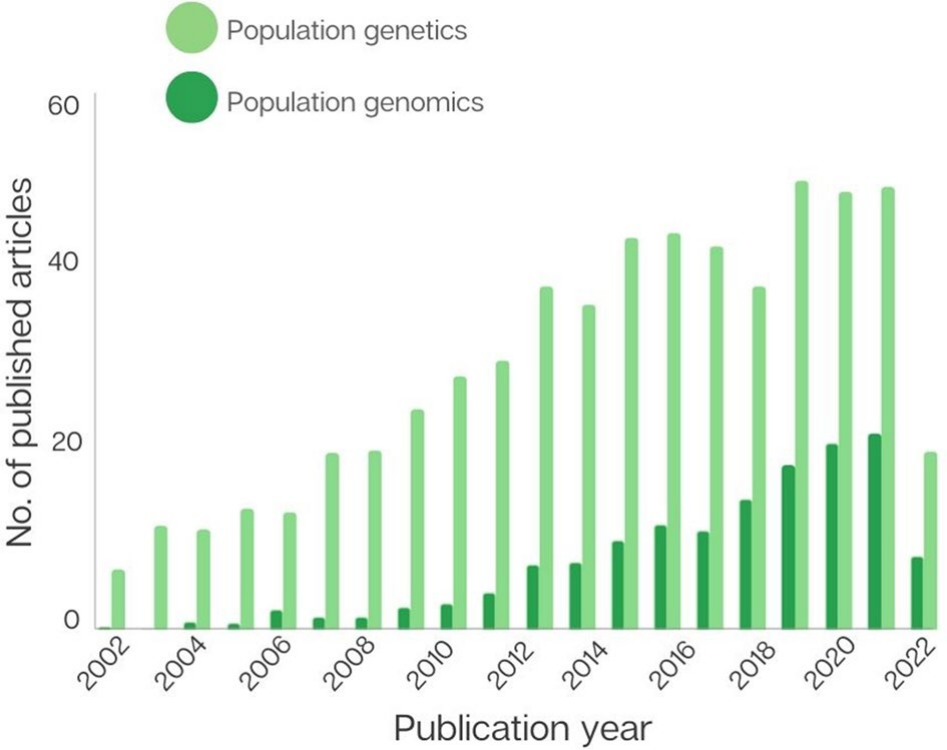
Number of published articles that apply “population genomics” (dark green) and “population genetics” (light green) in an invasive context over time (see Methods for details).

We searched across two academic databases to examine the number of published articles that target genomics, population genetics, and/or population genomics of invasive species. We found that publications utilising genetic markers (e.g. mtDNA, microsatellites, allozymes, amplified fragment length polymorphisms/AFLPs and/or SNPs) largely dominate invasive biology research (n = 3128), despite an increasing focus towards population genomics over the last ~ 20 years (Fig. 1). In 2011, genomics-based research made up just 9% (15 out of 165) of population studies on invasive species conducted that year; ten years later this figure had increased to 31% (116 out of 378). In fact, 15% (116 out of 779) of articles targeting population genomics of invasive species were published in 2021 alone. The escalation of population genomic data in the literature correlates with the decreasing cost of next generation sequencing (from $USD5292.39 in September 2001 to $USD0.006 in August 2021 per Mb).

### Population genetic data is largely available for invasives, but limited in scope

**Figure 2:**
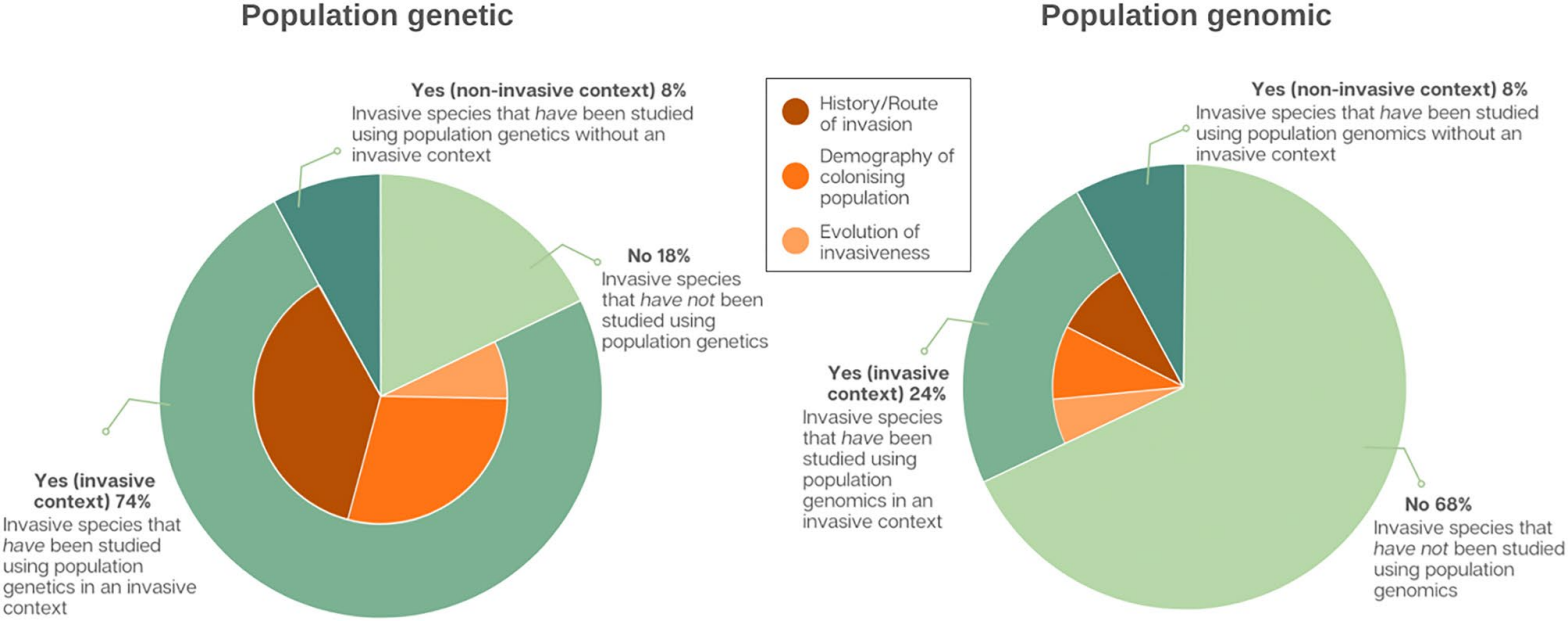
Proportion of invasive species from the WAS List (n = 100) for which researchers have utilised population genetic and population genomic data as a research tool.

As of May 2022, 82% of the WAS List species had been examined using some form of population genetic data (Fig. 2). Of 807 retrieved studies, at least one publication utilised genetic data in an invasive context for 74 (90%) of the examined species. These invasion-focused population genetic studies dominantly targeted the history/routes of incursion (51%) and the demography of colonising populations (39%), while the evolution of invasiveness has only rarely been examined using population genetic data (10%) (Fig. 2; Supplementary Information).

### Population genomics data for invasives is predominantly absent

Despite the encouraging pattern outlined above, we found that only 32% of the WAS List species had at least one publication that utilised population genomic data as a research tool. Thus roughly two-thirds of globally important, highly invasive species on the WAS List currently lack publicly-available population genomic data (Fig. 2). Of the 32% of species for which population genomic data is available, this data has been applied in an invasive context for the majority (75%), though this represents a total of just 24 of the 100 listed species. These population genomics-focused studies predominantly targeted the history/routes of incursion and the demography of colonising populations in similar proportions (~ 38%), while the evolution of invasiveness has received the least focus (24%) (Fig. 2; Supplementary Information).

### The study of invasives is subject to a limited geographical distribution of resources

We extracted the origin country of research organisations affiliated with the authors of each publication to investigate the likely geographical distribution of invasive genomic resources (e.g. tools and funding). Of the 809 articles that used population genetic data to study invasive species, author country of origin records (n = 1140) indicated that the top five countries are higher income countries: United States (n = 242), France (n = 118), Australia (n = 71), Germany (n = 70), and Spain (n = 67). While a smaller number of publications had author country of origins from lower-income countries, there were never more than 10 (i.e. < 1% of the total) publications per country. The United States also dominated the author country of origin records for the 91 articles that included population genomic data as a research tool to study invasive species, making up 49 of the 239 total records. In the top ten author countries of origin for the population genomic inavasion-focused data, Australia (n = 12) was again the only country in the Southern Hemisphere represented, and no countries from Africa were present (Supplementary Information).

Meanwhile, the majority (86%) of the total articles that were identified as having a population genomic context were published within an open access framework. This contrasts with our population genetic analysis, where less than half (41%) of the articles were open access. There was no significant relationship between geography and a presence or lack of open access publishing for either population genetic or population genomic publications (Supplementary Information).

### Invasive species commonly lack reference genomes

**Figure 3:**
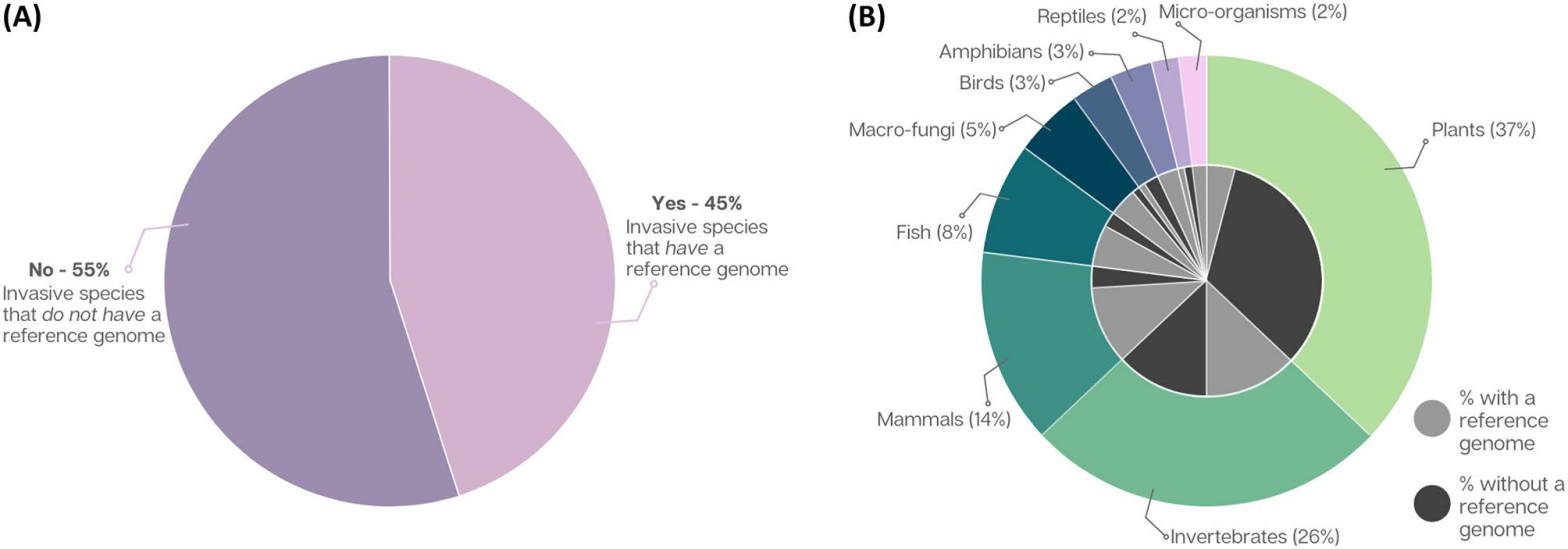
Proportion of invasive species from the WAS List (n = 100): (**A**) for which researchers have deposited a reference genome to NCBI; and (**B**) that correspond to the indicated taxonomic groups (outer circle), the associated proportion for which have (light grey) or lack (dark grey) reference genomes (inner circle).

We examined the National Centre for Biotechnology Information (NCBI) database and found that 45% of the WAS List species had a publicly-available reference genome (Fig. 3A). The WAS List is largely dominated by plant species (n = 37), followed by invertebrates (n = 26), and mammals (n = 14) (outer ring, Fig. 3B). However, mammals are disproportionately over-represented in terms of available reference genomes (78.6%). Plants are conversely under-represented, with ~ 89% of the WAS List plant species lacking genomic resources. Meanwhile, two of the three birds from the list lack reference genomes entirely, as do half of the list’s 26 invertebrate species (Fig. 3B).

## Discussion

We investigated the extent to which population genetic and genomic data have been used to study globally invasive species from the WAS List. We found that genetic data, as opposed to genomic data, is used more widely as a tool to study population dynamics of invasive species, though this is mainly limited to elucidating invasion history such as identifying routes of colonisation and source populations. Despite this, we found that publications relating generally to biological invasions and genomics (including population genomics) are gaining momentum in the literature, showing an increasing trajectory over the past ~ 20 years that aligns well with the reducing costs of next generation sequencing. However, only 32% of species on the WAS List have currently been studied in a population genomics context, and there is a large depauparacy (55%) of reference genomes that are available for these species in the commonly-used NCBI genome repository.

Recent studies exemplify the value of population genomic resources as tools for informing, monitoring, and managing biological invasions^30^. For example, whole genome scans of the predatory Northern snakehead fish, *Channa argus*, were used to identify the source population of invasions in parts of the United States for future prohibition of accidental and deliberate introductions^43^ and whole genome resequencing data has led to more targeted management of glyphosate resistance in populations of the weed, *Amaranthus tuberculatus*^44^. Despite this, Rius et al*.*^39^ found that just 33% of 117 published studies applying next-generation sequencing to invasive species between 2008 and 2015 had an invasive context. Similarly, we found that only 32% of species on the WAS List have been studied in a population genomics context, though we note that the list of Rius et al*.*^39^ included only 13 species from the WAS List. Within the 32% of species on the WAS List that had been studied in a population genomics context in our study, an encouraging majority (n = 24; 75%) focused on invasive objectives, however the least targeted aspect of invasion biology in these studies was the evolution of invasiveness. Although genome sequencing has been used in only a handful of invasive studies and a small number of organisms to date^30,40^, there is accumulating evidence that genetic changes contribute to invasion success^21,45^, so the underutilisation of population genomics for detecting the genomic architecture of invasion is disappointing. Comparing genomic divergence between invasive and native-range populations of the same species in particular holds great promise for elucidating and predicting the role of the genome in biological invasion^39^—an area that is clearly still yet to gain great traction.

Despite a variety of genome-generating initiatives (e.g. the Earth Biogenome Project: https://www.earthbiogenome.org/), under half (45%) of species on the WAS List currently have accessible reference genomes. However, this represents a sizable increase in the last three years, with McCartney et al*.*^40^ identifying 27/100 of species on the same WAS List as having reference genomes—a promising result on the surface that may indicate a rapidly growing investment in genomic resources. (This parallels the trend seen for the IUCN threatened species list, for which published genomes were available for 2.4% of the total 15,521 listed species as at January 2022—an increase from 0.8% in 2018^46^). However, reference genomes may be assembled for invasive species in a non-invasive context, e.g. the species may have high economic value, or high merit as a research model. Indeed, just 13 of the 27 species in McCartney et al*.*^40^ that had a reference genome had invasive status as an a priori rationale for genome assembly. In our case, species such as *Sus scrofa* (pig), *Oncorhynchus mykiss* (rainbow trout), and *Mus musculus* (field mouse) returned hundreds of documents in the literature searches, however, very few of these were relevant or translatable to invasion.

The lack of widespread application of genomic resources to invasion biology that we detect here is undoubtedly driven by the associated costs of generating such data. Financial and computational burdens, together with the required time and expertise, continue to place limits on the breadth and depth of genomic studies^19^, despite progress in technology, analysis pipelines, and bioinformatics training. Fortunately, many important questions in invasion biology can be addressed with fewer genetic markers and our population genetic results indicate that, although genomic approaches are superior in some instances, individual markers, such as mtDNA, still have an important ongoing role to play in invasive species research. However, a lack of equity in this space may explain our finding that most authors of invasive species research are predominantly based in higher-income countries, such as the United States and countries in Europe, rather than in locations in Africa or the Southern Hemisphere (Supplementary Information)—irrespective of whether the data was population genetic or genomic in nature (though we noted a slight increase in representation of lower-income author countries of origin in the population genetic versus genomic records, this never exceeded 1% of the total publications for these countries).

Mammals make up just 14% of the WAS List, including familiar species such as red deer, domestic cats, and stoats. However, they constitute roughly a quarter of the species that have a reference genome. Plants show the converse pattern, making up 37% of the WAS List but having a reference genome for only four species. These findings do not reflect the relative impacts of each invasive group (e.g.^47,48^); rather, taxon-specific idiosyncrasies likely play a role for some groups. For example, ploidy in plants can increase the complexity and challenge of genomic analysis compared to other taxonomic groups^49^ and amphibians have large and highly heterozygous genomes^50^. Fortunately, recent technological advancements, especially relating to long-read sequencing, are making genomic research more accessible and accurate, particularly for organisms with large and/or complex genomes^51^.

Of course, it is possible to learn a great deal about a species’ evolutionary properties without the use of a reference genome^52^. Such approaches are particularly useful for studying non-model species, but reduced genome complexity and potentially high degrees of missing data^52,53^ make them unsuitable for addressing certain study questions (e.g. genomic rearrangements^54^), while access to a reference genome can make answering other questions more efficient^40^. The lack of reference genomes identified here limits the resolution of genomic studies available for invasive species on the WAS List. However, several recent initiatives aim to sequence genomes of pests and/or pathogens (e.g. Ag100Pest Initiative: http://i5k.github.io/ag100pest; Plant Pathogen ‘Omics Initiative: https://bioplatforms.com/projects/plant-pathogen-omics/) and we argue that more funding, effort, and expertise should be allocated to such projects, particularly for the taxa that we have identified as having received little research attention, such as plants.

The limited taxonomic scope of invasive species from the WAS List that have received population genomic attention to date likely represents a broader limiting of evolutionary understanding of invasive species that is required to predict and prevent future incursions. Generally, the incorporation of population genetic research into policy decisions is becoming more widely adopted—particularly in its use for identifying invasion routes and clarifying taxonomic uncertainties prior to management^31,55,56^. However, incorporation of population genomic data into such policy has been minimal (a similarly slow translation of population genomics findings to applied wildlife conservation is also common^57^) despite its clear advantage over genetic data in many scenarios, as outlined here. As invasions are predicted to increase in frequency and magnitude with climate change, the implications of this will affect pest management at a global scale and, although the highest number of invasive species are found in developed nations, their threat to developing nations, where there are less resources available for invasion management, is much higher^1^.

Population genomic data and methods are revolutionising the field of biology and have the potential to change the way we study invasive organisms and accelerate the pace at which we can ultimately apply genomic resources to a policy and management setting. However, while genomics and population genomics are gaining momentum in invasive species research, there is much to be done. First, reference genomes need to be assembled and made publicly available for the vast proportion of invasive species that lack them, including those on the WAS List—we need to see more, targeted ‘invasomics’ reference genome initiatives. Second, more research should target population genomic analysis of invasive species, allowing for a greater understanding of the demo-genetic factors and intrinsic genetic mechanisms that lead to invasion success. This will aid in the development of proactive responses against invasive species that take a genome-informed approach to exploit specific species weaknesses to prevent their spread and limit their impact. Third, much of this research is cutting edge and, although 68% of species from the WAS List are yet to be studied with population genomic methods, over half of those that have been were published in the last 5 years. Further genomic uptake in this space should be maintained to ensure that genomic insights into invasive species continue at a pace that meets the escalating demands imposed by future climate change. In conjunction, the accessible nature of at least some of the population-based genomic data that has not currently been applied in a population genomic context could be retrospectively analysed with appropriate bioinformatic techniques to address invasive questions.

## Methods

### IUCN “100 of the world’s worst invasive alien species”

The International Union for Conservation of Nature (IUCN) is an organisation of governments, civil society organisations, and experts perhaps best known for publishing the ‘Red List of Threatened Species’, which provides a comprehensive index of the conservation status of species worldwide and their associated risk of extinction. The Invasive Species Specialist Group (ISSG) is a network of experts and policy makers organised under IUCN that aims to increase awareness of invasive species and their impact on the environment, as well as blueprint prevention, management, and/or eradication plans^58^. The Global Invasive Species Database (GISD) is a product of the ISSG, developed by Clout and Lowe^59^ to aid the early detection and management of invasive species in developing countries. The ‘100 of the World’s Worst Invasive Alien Species’ list (‘WAS List’, hereafter) was first published in 2000 for both scientific and communication purposes (e.g.^17,60^). Species on the list are chosen based on their impact on biodiversity and human activities, as well as their illustration of issues surrounding biological invasion and representation of a diverse selection of taxonomic groups, from microorganisms to plants and vertebrates^61^.

### Database searches

We used database searches in our analyses, with all methods carried out in accordance with relevant guidelines and regulations. Web of Science and PubMed searches were performed (May 2022) to examine: the uptake of ‘population genetics’ and ‘population genomics’ analysis in an invasion biology context, and the degree to which population genetic, genomic, and/or reference genome resources exist for each of the WAS List species.

In the first search, the terms (“population genetic*” OR “next generation sequencing” OR “SNP*” OR “single nucleotide polymorphism*” OR allozyme* OR AFLP* OR microsatellite* OR mtDNA OR “mitochond* DNA” OR “nuclear DNA") AND (“invasive” OR “weed” OR “pest”) AND (“animal*” OR “species” OR “organism*”) were applied to titles and abstracts in the Web of Science and PubMed databases, yielding a total of 3276 results. Publication years for each search were obtained using the Web of Science ‘analyse results’ tool. To identify differences between population genetic and population genomic trends though time, this was followed by a second search using the terms (“population genomic*” OR “next generation sequencing” OR “SNP*” OR “single nucleotide polymorphism*”) AND (“invasive” OR “weed” OR “pest”) AND (“animal*” OR “species” OR “organism*”), which returned 779 results.

In a separate search across both databases, keywords for each species associated with the WAS List were used to establish whether: (a) population genetic; and (b) population genomic data was available for inferring evolutionary patterns and processes. The keyword string used for each species and search was: (a) (“common name*” OR “species name”) AND (“population genetic*” OR “next generation sequencing” OR “SNP*” OR “single nucleotide polymorphism*” OR allozyme* OR AFLP* OR microsatellite* OR mtDNA OR “mitochond* DNA” OR “nuclear DNA") and (b) (“common name*” OR “species name”) AND (“population genom*” OR “next generation sequencing” OR “SNP*” OR “single nucleotide polymorphism*”); and titles and abstracts were searched in each case. For (a), this search yielded 0–535 results per species, and 4399 articles were retrieved overall. For (b), the search yielded from 0 to 258 results per species, and 1217 total articles were retrieved. The relevance of each document for these searches was determined based on a screening of the abstract, resulting in the removal of articles that did not contain: samples from wild individuals, samples from different populations, and for (b) samples that lacked a focus on genome-wide data. For (a) and (b), if at least one abstract contained data and terminology relevant to population genetics or genomics (e.g. population structure, gene flow/genetic drift, genetic diversity, phylogeography), then the species was considered ‘positive’ for either data type and was further examined and scored for invasive context—in this case, each study was evaluated and scored for its *dominant* research focus: the history or route of incursion, the demography of the invading population, or the evolution of invasiveness. For both (a) and (b), metrics such as year of publishing, origin country of the research organisations affiliated with each author, and publication availability (i.e. open access status) were collected for each species using the Web of Science ‘analyse results’ tool.

### NCBI searches

The National Centre for Biotechnology Information (NCBI) database was used to track whether each species on the WAS List had a publicly-available reference genome associated with it. Although there are likely other public repositories for genomic data, NCBI contains the largest bank of molecular biological and genetic data available and its genome database contains the most up to date sequence and mapping data for a range of organisms^62^; as a result, we feel it best captures the most publicly accessible genome data available. In May, 2022 the scientific name of each of the 100 species was entered into the search bar of the NCBI website (https://www.ncbi.nlm.nih.gov/) with the database category set to ‘genome’. If the resulting search indicated that there was a reference genome, that species was recorded as ‘positive’ for this data type.

## Supplementary Information

Supplementary Information.

## Data Availability

All data generated or analysed during this study are included in this published article [and its Supplementary Information files].
